# 475. The Utility of Community-Academic Partnerships in Promoting the Equitable Delivery of COVID-19 Vaccines in Black Communities

**DOI:** 10.1093/ofid/ofab466.674

**Published:** 2021-12-04

**Authors:** Jacinda Abdul-Mutakabbir, Samuel Casey, Veatrice Jews, Andrea King, Kelvin Simmons, Bridgette Peteet, Juan Carlos Belliard, Michael Hogue, Ricardo Peverini

**Affiliations:** 1 Loma Linda University School of Pharmacy, Redlands, California; 2 Congregations Organized for Prophetic Engagement, San Bernardino, California; 3 Inland Empire Concerned African American Churches, San Bernardino, California; 5 Loma Linda University School of Psychology, Loma Linda, California; 6 Loma Linda University School of Public Health, Loma Linda, California; 7 Loma Linda School of Medicine, Loma Linda, California

## Abstract

**Background:**

In the U.S., non-Hispanic Black individuals are disproportionately represented amongst COVID-19 mortalities. The COVID-19 vaccines are poised to change this outcome; however, inequitable access and decades of medical mistreatment have resulted in healthcare mistrust and an associated low uptake within this group. Loma Linda University (LLU) houses the largest mass vaccination site in San Bernardino County (SBC) California; nevertheless, there has been a perpetual low representation of Black vaccinees. To increase the number of Black persons vaccinated, a selected team at LLU leveraged a community-academic partnership model to address vaccine hesitancy and increase access to the COVID-19 vaccines. The objective of this study was to evaluate the number of Black persons vaccinated in community settings compared to the mass clinic.

**Methods:**

LLU developed a tiered approach to increase COVID-19 vaccinations within Black SBC communities. The first tier engaged faith leaders with the academic community in disseminating COVID-19 health information, the second included culturally representative LLU healthcare professionals in the delivery of COVID-19 educational webinars, and the third was to conduct low barrier, remote-site vaccination clinics, within targeted Black communities. Following these efforts, we compared the number of Black individuals vaccinated in the LLU mass clinic to those vaccinated in the community remote-site clinics.

**Results:**

The remote-site COVID-19 vaccination clinics commenced in February 2021. From February 1 until April 30, 2021, 24,808 individuals were vaccinated in the LLU mass clinic with a first dose (Pfizer or Moderna) or single dose (Janssen) of a COVID-19 vaccine, however, only 908 (3.7%) were Black vaccinees. Contrastingly, the LLU remote site clinics vaccinated 1,542 individuals with a first or single dose of a COVID-19 vaccine. Of those vaccinees, 675 (44%) were Black.

**Conclusion:**

The multi-tiered community approach (remote-site vaccination clinics) resulted in a necessary overrepresentation of Black vaccinees, previously underrepresented in the LLU traditional mass vaccination clinic effort (44% vs. 3.7%, respectively). Further research is warranted to examine the key elements to increase vaccinations amongst minoritized groups.

COVID-19 Vaccination Comparisons Between Models

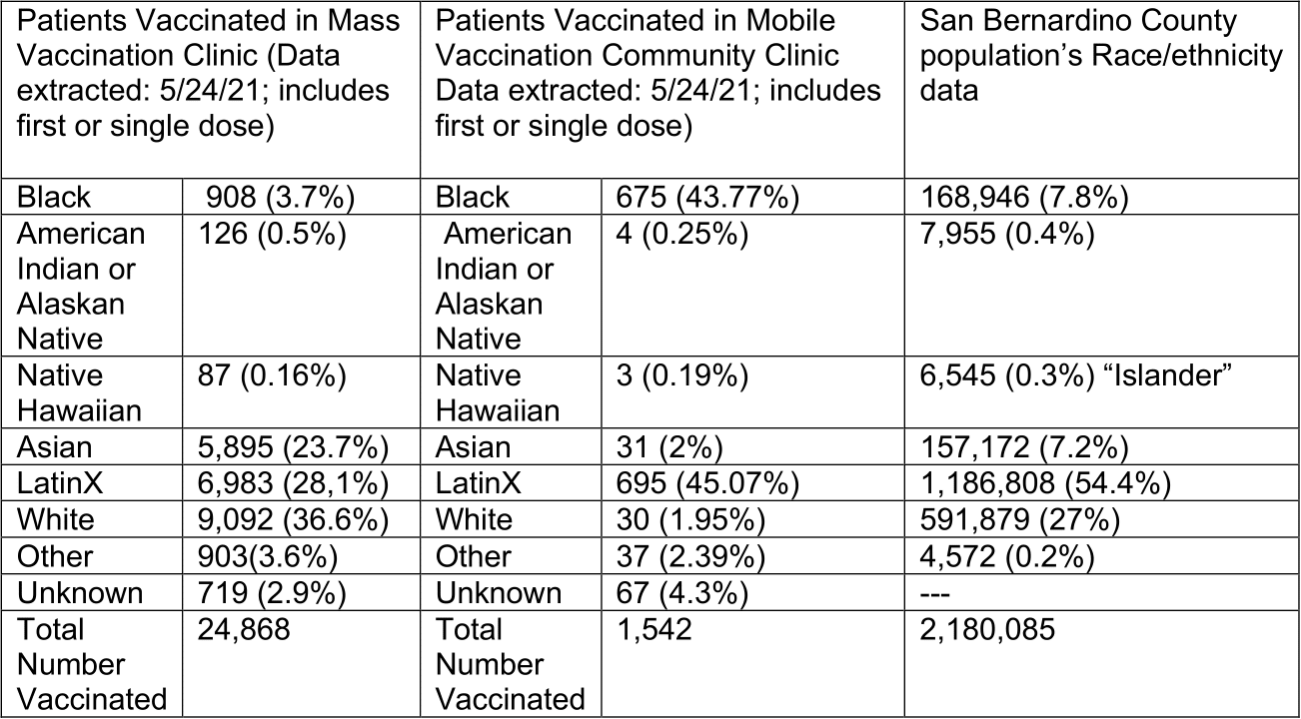

This table includes data from the Loma Linda University Mass Vaccination Clinic and the Remote-Site Vaccination Efforts compared to the San Bernardino County Demographics

**Disclosures:**

**All Authors**: No reported disclosures

